# Transcriptome Profiling after Early Spinal Cord Injury in the Axolotl and Its Comparison with Rodent Animal Models through RNA-Seq Data Analysis

**DOI:** 10.3390/genes14122189

**Published:** 2023-12-08

**Authors:** Juan Carlos González-Orozco, Itzel Escobedo-Avila, Iván Velasco

**Affiliations:** 1 Instituto de Fisiología Celular-Neurociencias, Universidad Nacional Autónoma de México (UNAM), Mexico City 04510, Mexico; gorozco@ifc.unam.mx (J.C.G.-O.); iescobed@ifc.unam.mx (I.E.-A.); 2 Laboratorio de Reprogramación Celular, Instituto Nacional de Neurología y Neurocirugía “Manuel Velasco Suárez”, Mexico City 14269, Mexico

**Keywords:** spinal cord injury, neural regeneration, regenerative medicine, transcriptomics, axolotl

## Abstract

Background: Traumatic spinal cord injury (SCI) is a disabling condition that affects millions of people around the world. Currently, no clinical treatment can restore spinal cord function. Comparison of molecular responses in regenerating to non-regenerating vertebrates can shed light on neural restoration. The axolotl (*Ambystoma mexicanum*) is an amphibian that regenerates regions of the brain or spinal cord after damage. Methods: In this study, we compared the transcriptomes after SCI at acute (1–2 days after SCI) and sub-acute (6–7 days post-SCI) periods through the analysis of RNA-seq public datasets from axolotl and non-regenerating rodents. Results: Genes related to wound healing and immune responses were upregulated in axolotls, rats, and mice after SCI; however, the immune-related processes were more prevalent in rodents. In the acute phase of SCI in the axolotl, the molecular pathways and genes associated with early development were upregulated, while processes related to neuronal function were downregulated. Importantly, the downregulation of processes related to sensorial and motor functions was observed only in rodents. This analysis also revealed that genes related to pluripotency, cytoskeleton rearrangement, and transposable elements (e.g., *Sox2*, *Krt5*, and *LOC100130764*) were among the most upregulated in the axolotl. Finally, gene regulatory networks in axolotls revealed the early activation of genes related to neurogenesis, including *Atf3/4* and *Foxa2*. Conclusions: Immune-related processes are upregulated shortly after SCI in axolotls and rodents; however, a strong immune response is more noticeable in rodents. Genes related to early development and neurogenesis are upregulated beginning in the acute stage of SCI in axolotls, while the loss of motor and sensory functions is detected only in rodents during the sub-acute period of SCI. The approach employed in this study might be useful for designing and establishing regenerative therapies after SCI in mammals, including humans.

## 1. Introduction

Spinal cord injury (SCI) is a disturbing event that causes profound changes in the structure and function of this organ. Moreover, depending on the type of injury and the extent of the damage, these changes often result in temporary or permanent sensory and motor disabilities in the individuals who suffer from it [1,2]. Traumatic injury due to external physical impacts (e.g., falls or car accidents) is the leading cause of SCI; nonetheless, SCI can also occur from non-traumatic events, such as tumor development or inflammatory processes derived from infectious conditions [3,4]. The pathophysiological events of traumatic SCI are temporally separated into the acute (<2 days), sub-acute (2–14 days), intermediate (14 days to 6 months), and chronic (>6 months) phases [1]. During the acute phase, the initial damage induces neuronal death and an acute inflammatory response, that in turn induces glial scar formation during the subsequent phases. Glial scarring, together with the null capacity of neuronal turnover in mammals, drastically reduces the spinal cord recovery potential [5], triggering, in most cases, the neurological deficits that are observed in patients with chronic SCI, such as loss of motor control and alteration of sensation [6]. In the United States of America, it is estimated that approximately 450,000 people are permanently disabled due to traumatic SCI [7]; in Western Europe, the estimated incidence of traumatic SCI is 15 cases per million people, while the overall global incidence corresponds to 10.5 cases per 100,000 persons, with 768,473 new cases each year around the world [1,8].

Animal models for traumatic SCI, typically in rodents, have provided significant information about the pathological processes that occur, as well as how recovery could be promoted after the insult [9]; however, to date, there is no effective therapy that reverses the histological changes induced in the damaged spinal cord, neither is there a clinical strategy capable of promoting the regeneration of the damaged neural circuits.

The axolotl is a salamander with an exceptional regenerative capacity. After tissue damage or amputation, axolotls can repair and replace entire anatomical parts, including several regions of the brain and spinal cord, restoring all the neuronal and glial cell types observed before damage [10,11]. Therefore, to comprehend the molecular differences with other vertebrate species that do not exhibit regeneration, such as mammals, several studies conducted to understand the regenerative capacity of the axolotl have focused on the identification of genes and molecular processes that lead to the repair and replacement of damaged tissues [12,13].

By analyzing RNA-seq data obtained from public datasets, in this study, we compared the transcriptomic responses of the spinal cords in axolotls and rodents after traumatic injury, to determine the gene expression differences between these species during the first days post-injury (dpi). With this approach, we aimed to identify transcriptional programs that allow the regeneration of the spinal cord in axolotls, as well as to understand the molecular processes that inhibit the repair of the damaged spinal cord in mammals. Such analysis could provide clues for therapeutic targets to be applied in regenerative medicine.

## 2. Materials and Methods

### 2.1. Data Collection

After a preliminary screening, three RNA-seq datasets derived from different species (axolotl, rat, and mouse) with traumatic SCI were analyzed and compared in this study. Datasets were collected from the European Nucleotide Archive (ENA) [14] under the following accession numbers: axolotl [PRJNA378982] [15], rat [PRJNA760277] [16], and mouse [PRJNA193596] [17]. The specifications of each dataset are described in Table 1.

### 2.2. Data Pre-Processing

The quality of the obtained raw sequence reads in FASTQ format from the RNA-seq datasets were verified using FastQC (https://www.bioinformatics.babraham.ac.uk/projects/fastqc/, accessed on 18 April 2023). Then, the sequencing datasets were aligned with their respective reference genomes using the program HISAT2 [18]. For axolotl, the reference genome was AmexG_V6.0-DD [19], while for rat and mouse were mRatBN7.2 (GenBank assembly accession: GCA_015227675.2) and GRCm39 (GCA_000001635.9), respectively. The resulting SAM files from the alignments were converted to BAM files using SAMtools (ver. 1.18) [20]. GTF annotations files were used to tag aligned reads using the R package Rsubread (ver. 2.16) [21]; a data matrix containing read counts was extracted using the function featureCounts also from Rsubread. For axolotl genes, gene names annotated also in rat or mouse GTF files were preferred, otherwise, gene names also annotated in other species with better genome description were used. If neither was present, the axolotl gene IDs from the current reference genome (Amex60DD) were used as the gene names. To complete three technical replicates from the axolotl data, bootstrap resampling was applied to the matrix read counts in R [22].

### 2.3. Differential Gene Expression Analysis 

The matrices obtained in Rsubread containing the read counts (Table S1) were used as input for gene expression analyses. Differential expression of three groups (intact, 1–2 dpi, and 6–7 dpi; *n* = 3 for each condition) from each species was analyzed in R by using the package DEseq2 [23]. Read counts were normalized by library size and gene counts were modeled by negative binomial distribution. Differentially expressed genes (DEG) were detected by the Wald test using a threshold of false discovery rate (q-value) < 0.005 and a fold-change <−2 for downregulated genes and >2, for upregulated genes. Heatmaps were plotted with the R function pheatmap. Volcano plots, Venn diagrams, and bar plots were also plotted from DEG in R. Reactome and KEGG pathways were derived from DEG identified in heatmaps and Venn diagrams, respectively, using the package enrichR (ver. 3.2) setting a q-value of 0.005.

### 2.4. Gene Ontology Analysis 

DEG obtained from DEseq2 was used to perform gene ontology (GO) enrichment analysis using the R package clusterProfiler (ver. 4.10) [24] for each time post-injury. GO annotations for biological processes were queried by gene symbols using the organism database packages (OrgDb) for rat and mouse. For axolotl, the human Org.db was used using the gene symbols identified as human orthologs provided in the axolotl reference genome. Benjamini–Hochberg correction (BH) was used for false discovery rate estimation during GO analysis while the cutoff for q-value was 0.005. Bar plots were used to visualize the top GO terms enriched by the upregulated and downregulated genes for each time post-injury. Gene regulatory networks showing the result of the enriched genes for representative GO annotations were plotted using the cnetplot function from the clusterProfiler package.

### 2.5. Code Availability

The code employed in this study for gene expression analysis is available on GitHub at link https://github.com/JCGO221/Axolotl_SCI_Comparatives/ (accessed on 26 November 2023).

## 3. Results and Discussion 

### 3.1. Changes in the Global Gene Expression after Traumatic SCI Show Large Differences between Axolotls and Rodents

**Figure 1:**
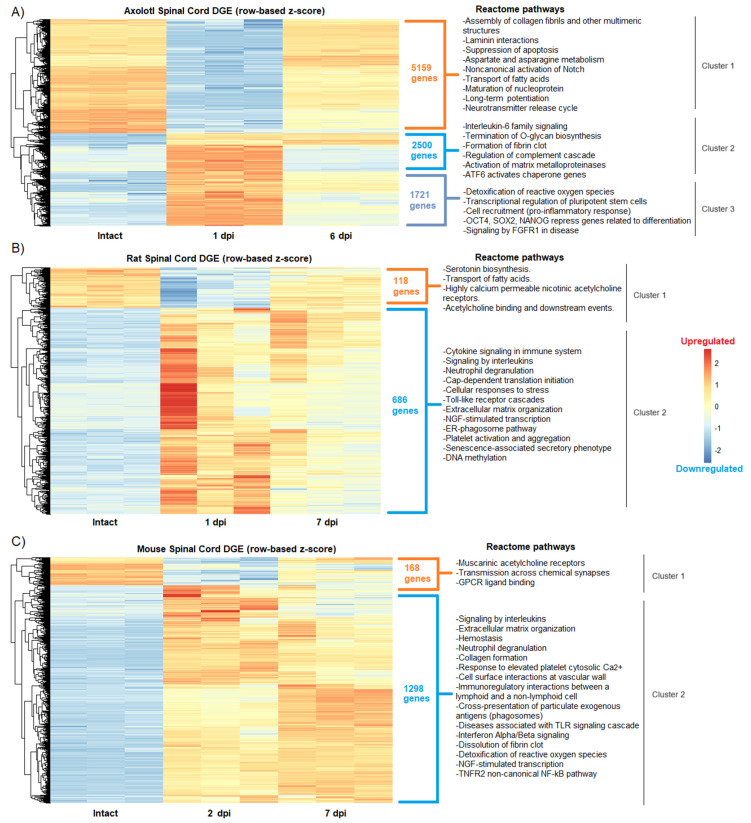
Changes in the global gene expression during acute and sub-acute SCI phases in axolotl and rodents. Heatmaps show the differential gene expression (DGE) categorized by hierarchical clustering in (**A**) axolotl after 1 and 6 dpi; (**B**) rat after 1 and 7 dpi; and (**C**) mouse after 2 and 7 dpi. The intact spinal cord condition is shown for all species. Reactome pathways detected for each gene cluster in all species are depicted.

RNA-seq data from axolotls, rats, and mice with acute (1–2 dpi) or sub-acute (6–7 dpi) SCI were analyzed to determine changes in global gene expression after traumatic injury. First, and as was expected from published works [25,26,27], we observed in the hierarchical cluster analysis similarities, but also significant differences, in groups of genes that were upregulated and downregulated between the intact and SCI groups for the three species analyzed (Figure 1). 

Particularly in axolotl, we found a cluster of genes (cluster 2) that were activated in the acute phase and that drastically decreased their expression levels in the sub-acute period, while others remained strongly upregulated (Figure 1A), an event that was not noticed in rodents. Furthermore, the enrichment analysis to depict Reactome pathways for each gene cluster showed in axolotl the upregulation of genes related to pluripotent stem cells and self-renewal, including *Oct4*, *Sox2*, and *Nanog* [28] at 1 dpi, as well as the enrichment of pathways associated to signaling of FGFR1, detoxification of reactive oxygen species (cluster 3,) and control of the quality of the proteins in response to stress by upregulation of ATF6 (cluster 2) (Figure 1A). Interestingly, ATF6 has been related to early neurodevelopment and the onset of myelination in mammals [29], while FGFR1 ligands have been proposed to restore spinal cord function after an insult [30]. 

These results demonstrate that protective and stem cell-related mechanisms are activated in axolotls very early in response to SCI, and not in rodents. Moreover, it was detected that neural functions associated with the activity of acetylcholine receptors were impaired in both rats and mice after injury (cluster 1 in both species) (Figure 1B,C), while in axolotl the downregulation of such functions was not detected by Reactome pathway analysis. 

Nevertheless, molecular similarities were also identified among the studied species, particularly in the extracellular matrix and immune processes that were upregulated upon SCI. In agreement, a previous study where comparative transcriptomics between rats and axolotls after SCI was performed through microarray analysis, it was found that multiple extracellular matrix genes were upregulated in both species, thus highlighting the importance of extracellular matrix remodeling in response to SCI [31]. Despite this, and as was expected, the most common regulated processes between rodents and axolotls were those related to the activation of the immune system, particularly the activation of signaling by interleukins (cluster 2 in rats and mice), whose function has been extensively reviewed in SCI [32]; notably, interleukin-6 signaling was specifically detected in axolotl (cluster 2), which has recently been reported that its overexpression in rodents with SCI can substantially increase their functional recovery [33]. 

**Figure 2:**
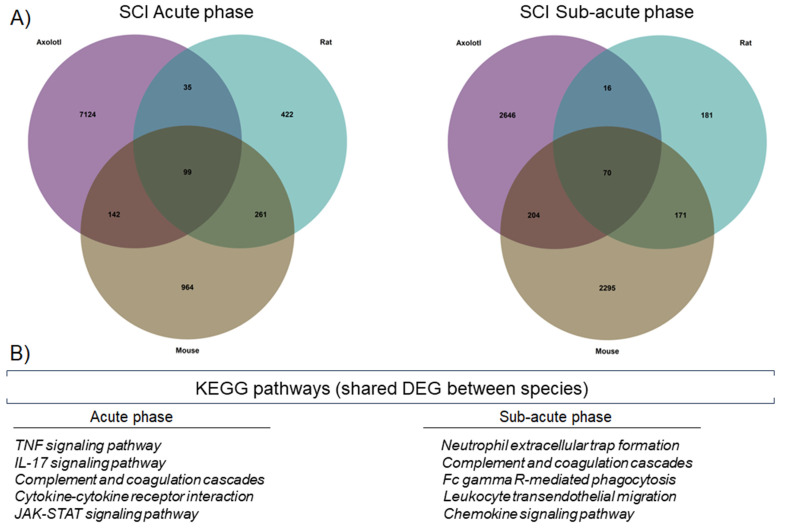
Axolotls and rodents share specific molecular changes after SCI. (**A**) Venn diagrams for DEG in axolotl, rat, and mouse, during the acute and sub-acute phases of SCI. (**B**) KEGG pathways were detected by enrichment analysis of the shared DEG between the studied species.

To further corroborate whether there were similar sets of genes that were regulated in the three species, at each stage of SCI analyzed, a Venn diagram was drawn up. Thus, we observed that 99 genes were shared in the acute stage, while 70 genes were shared during the sub-acute stage (Figure 2A). Importantly, the KEGG pathways detected for the genes shared between the species corresponded to immunological processes, indicating again that in axolotls and rodents, there are common genes and molecular pathways that are activated shortly, in response to SCI (Figure 2B).

Finally, DEG and cluster analysis also revealed that an elevated number of genes are significantly regulated in axolotl after SCI in comparison to rodents (Figures S1–S3), suggesting that a strong response at the level of gene expression is a trait of SCI in this species. This finding might be attributed to the size of the axolotl genome, which is much larger than that of rodents. Moreover, the axolotl genome is currently the largest ever sequenced and has a high number of repeated sequences product of transposon insertions, which are proposed to have some participation in the regeneration capacity of this animal [15,19]. 

### 3.2. Top Regulated Genes in Axolotl and Rodents in the Acute and Sub-Acute Stages of SCI

**Figure 3:**
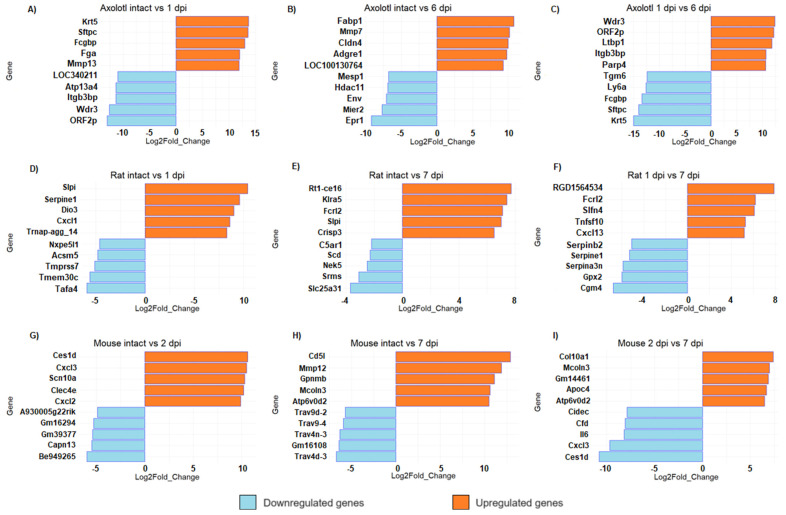
Top list of upregulated and downregulated genes in axolotl (**A**,**D**,**G**), rat (**B**,**E**,**H**), and mouse (**C**,**F**,**I**) with acute or sub-acute SCI. For all comparisons, the represented change corresponds to the second condition, relative to the first. All panels show bar plots with the most upregulated and downregulated genes for each animal at the indicated timepoint according to its fold change (q-value < 0.005).

Unlike mammals, axolotls can fully regenerate the spinal cord after injury [11,34]. However, given the number of genes regulated in axolotl, it is interesting to analyze the molecular circuitry that is triggered early after SCI and that is required to initiate a pro-regenerative response during subsequent stages of SCI in this species. Then, to narrow the list of interesting genes, we performed a top list of DEG (Figure 3). We found that keratin 5 (*Krt5*) was the most upregulated gene in axolotl at 1 dpi (Figure 3A). Keratin intermediate filaments are key components of the cytoskeleton, they support cellular rigidity and stability and are also associated with cell adhesion and migration; also, *Krt5* is a marker of basal and progenitor cells in mammalian epithelial tissue and has been identified as a regulator in the regeneration of limbs in axolotl; Randal Voss reported that it is highly upregulated during the 2–3 days post-amputation in forelimbs [35]. A gene related to blood clot (*Fga*), and genes related to the immune response and extracellular matrix organization (*Fcgbp* and *Mmp13*, respectively) were also detected among the most upregulated genes at 1 dpi in axolotl; particularly, it has been proposed that *Mmp13* may promote a more permissive environment for axonal regeneration in mice with SCI through its action on infiltrating monocytes [36]. 

Interestingly, *ORF2p* and *LOC340211* were detected among the top downregulated genes at 1 dpi, which are identified as genes of retroviral origin [37]. Meanwhile, at the sub-acute phase in axolotl (6 dpi), we found that *Fabp1* was the top upregulated gene (Figure 3B); it is important to mention that a gene from the same family of fatty acid binding proteins, *Fabp7*, regulates inflammation and has a neuroprotective role in mice with autoimmune encephalomyelitis [38], so it would be important to study the effects of *Fabp1* activation in rodents with SCI. Remarkably, *LOC100130764* was among the top upregulated genes at 6 dpi in axolotl, and it is identified as LINE retrotransposon element (https://www.ncbi.nlm.nih.gov/gene/100130764, accessed on 20 August 2023); moreover, Env gene, which is also of retroviral origin [39], was a top downregulated gene at this SCI phase. Thus, it is worth studying the potential role of highly repeated sequences of retroviral origin in the regeneration of the spinal cord of the axolotl, and determining if the diminished number of these genetic elements in the mammalian genome is a crucial factor for the absence of regenerative processes in these species.

In rodents, among the genes that were most upregulated in the acute stage (1–2 dpi) were the CXC motif chemokine ligand family—*Cxcl1*, *Cxcl2*, and *Cxcl3* (Figure 3D,G)—which are related to the inflammatory response, suggesting an acute immune response immediately after damage. It is known that CNS injury stimulates the expression of several proinflammatory chemokines and cytokines, including *Cxcl1*, and *Cxcl2*, which act to recruit leukocytes at lesion sites [40]. Interestingly, in rat, secretory leukocyte peptidase inhibitor (*Slpi*) was upregulated both in acute and sub-acute phases of SCI (Figure 3D,E). *Slpi* has anti-inflammatory properties, and it has been reported to promote wound healing [41]. Ghasemlou et al. reported in 2010 that Slpi has an early protective effect modulating the inflammatory response reducing NF-kB activation and TNF-a expression in the first few days after spinal cord injury [42]. Moreover, *Slp1* has been reported to increase dramatically between two and five days after SCI in humans [43], so it would be interesting to verify whether *Slp1* has also a protective effect in humans. Also, we found that *Mmp12* was a top-upregulated gene at 7 dpi in mice (Figure 3H). It is important to note that *Mmp12* has a negative effect on SCI in mice and contributes to the development of intermediate and chronic SCI [44]. 

Finally, the comparison between the acute and sub-acute phases in all species shows that some genes that were strongly upregulated during the acute phase are downregulated at the beginning of the sub-acute phase, and vice versa. For example, in axolotls, *Krt5* and *Sftpc* are upregulated in the acute phase and then they were significantly downregulated in the sub-acute phase; conversely, *Wdr5*, *ORF2p*, and *Itgb3bp* were downregulated in the acute response but significantly increased in the sub-acute timepoint. These results indicate that there is a switch in the transcriptome signature between post-injury phases, each one characterized by its own gene expression profile (Figure 3C,F,I). The complete list of all DEG in each organism for all timepoints is provided in Tables S2–S10.

### 3.3. Gene Ontology Analysis Shows Differences and Similarities in Biological Processes between Axolotl and Rodents after Traumatic SCI

**Figure 4:**
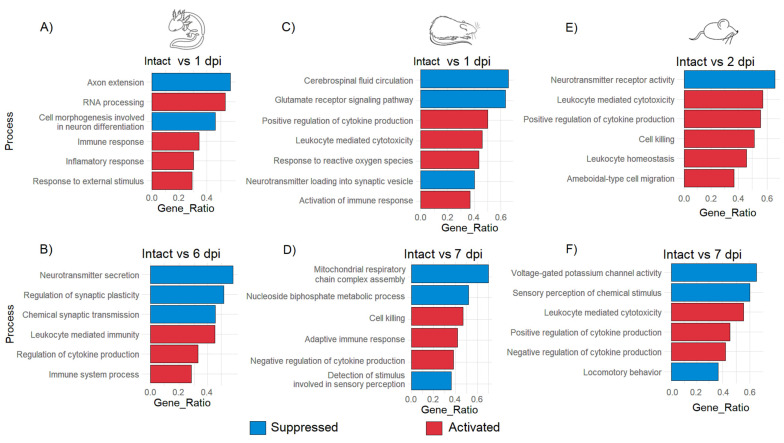
GO enrichment analysis of biological processes in axolotl, rat, and mouse during acute and sub-acute SCI. Enriched biological processes for axolotl at 1 dpi (**A**) and 6 dpi (**B**); rat at 1 dpi (**C**) and 7 dpi (**D**); mouse at 2 dpi (**E**) and 7 dpi (**F**). The false discovery rate was estimated by BH correction. The cutoff for the q-value was 0.005.

GO analysis was performed to detect the most enriched biological processes according to DEG at each timepoint after injury. After 1 dpi in the axolotl, it was observed that repair programs begin soon after the traumatic damage as indicated for the GO annotations “immune response” and “response to external stimulus” that were activated; these biological processes at this timepoint were also accompanied along with the annotation of “RNA processing”, which indicates again that a strong response at the level of gene expression is triggered after primary injury; meanwhile, “axon extension” and “cell morphogenesis involved in neuron differentiation” were suppressed at 1 dpi, suggesting an early activation of stem cells in the injury site as was observed in the hierarchical cluster analysis (Figure 4A and Figure S4A). 

Meanwhile, at 6 dpi, the suppression of the processes “neurotransmitter secretion” and “regulation of synaptic plasticity” was noticed in axolotls (Figure 4B). Regarding rat and mouse, it was detected that specific processes related to the acute immune response including “positive regulation of cytokine production”, “cell killing” and “leukocyte homeostasis” were predominant during the acute period of SCI, similar processes that were noticeable in the axolotl until the sub-acute phase. This finding was consistent with a previous study where it was shown that after a week, the injured spinal cord of the axolotl strongly upregulates genes related to the acute immune response, at the time in which visible regenerative tissue has been formed in the wound area [45].

Processes associated with neural functions like “cerebrospinal fluid circulation” or “neurotransmitter receptor activity” were immediately suppressed in rodents during the acute phase (Figure 4C,E); importantly, processes associated with the acute immune response were still observed during the sub-acute SCI in rodents. Also, the observed suppression of biological processes like “mitochondrial respiratory chain complex assembly” (Figure 4D) could suggest that induced oxidative stress is caused by the primary injury and that the acute immune response can increase the damage to the spinal cord in mammals instead of alleviating it. Moreover, the alteration of neurological functions was also noted in rat and mouse in the sub-acute phase since activities like “detection of stimulus involved in sensory perception”, “sensory perception of chemical stimulus”, or even “locomotory behavior” were suppressed (Figure 4D,F and Figure S4B,C). Therefore, these results indicate that the strong immune response triggered immediately by traumatic SCI in rodents might inhibit neurorepair mechanisms, and create a noxious environment that leads to the loss of the neurological functions associated with SCI starting at the sub-acute stage, while in axolotls a lax immune response from early phases of SCI could facilitate the triggering of molecular programs related to the repair and subsequent regeneration of the spinal cord. 

**Figure 5:**
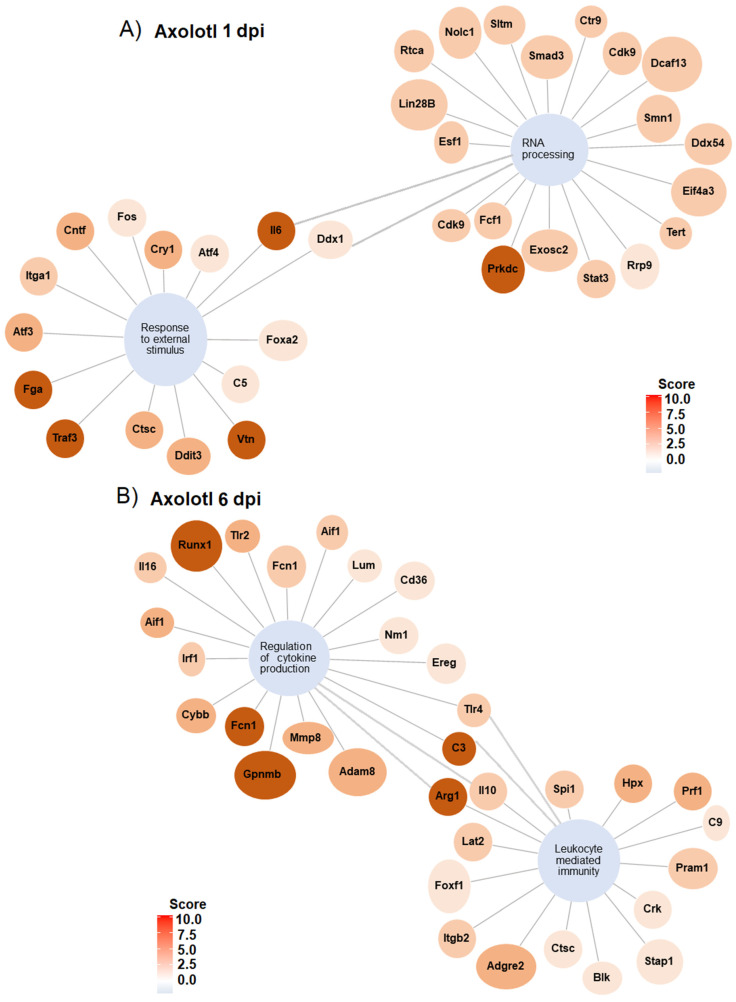
Gene regulatory networks for GO terms in axolotl with SCI at 1 and 6 dpi. (**A**) Gene networks for GO enriched terms “RNA processing” and “response to external stimulus” at 1 dpi. (**B**) Gene network for GO enriched terms “regulation of cytokine production” and “leukocyte mediated immunity” at 6 dpi.

To gain insights into the networks that lead to the biological processes regulated in response to SCI in axolotl and rodents, gene regulatory networks were plotted to identify specific genes that were associated with the mark-enriched GO terms. Genes related to “response external stimulus” and “RNA processing” in axolotl at 1 dpi are connected through *Il6* and *Ddx1*. Furthermore, detected genes for these GO terms include a few genes related to neurodevelopment and transcription/translation regulation (Figure 5A); for example, the gene *Atf4* was upregulated along the GO term “Response to external stimulus”, whose activity has been related to cortical neurogenesis in mammals [46]. Likewise, *Foxa2* is a gene associated with neuronal differentiation in mammals, particularly to dopaminergic differentiation during midbrain development. In addition, the genes *Vtn*, *Atf3*, and *Cntf* identified in our analysis are also related to the regulation of neurogenesis [47,48,49]; we propose to study all these genes to determine their potential role in spinal cord recovery in mammals. In addition, we found that the gene *Tert*, related to stem cells [50], was upregulated along the GO term “RNA processing”. 

The gene regulatory network at 6 dpi shows GO terms related to the immunological response after an injury during the sub-acute phase in axolotl; among the most upregulated genes for these terms are included *Runx1*, *Fcn1*, *C3*, *Arg1*, and *Gpnmb* (Figure 5B). Whereas the gene networks for representative GO terms in rodents during the acute and sub-acute periods of SCI show a strong regulation of genes associated with the immune response, including the upregulation of genes associated with cell clearance, inflammation, and mobilization of immune cells (Figures S5 and S6). 

Importantly, we identified that downregulation of the genes *Cox14*, *Uqcc1*, *Coa8*, *Dmac1*, or *Bcs1l* were related to the repression of the GO term “mitochondrial respiratory chain complex assembly” in rat, while the downregulated genes associated with “locomotory behavior” in mice were *Lmx1b*, *Grin1*, *Gad1*, *Nrg1*, *Chat*, *Dscam*, *Abat*, *Pak6*, *Fgf12* and *Hoxd9*. Thus, it would be truly important to study whether the activation of any of these genes could have any positive effect on the functional recovery of mammals with SCI. 

The importance of the immune system in regulating pro-regenerative responses in axolotl has been well documented during limb regeneration, where injury elicits an immediate wound healing response while in mammals promotes a strong innate immune response that triggers, in later phases of the injury, a fibrotic scarring program that limits the ability of the damaged tissue to regenerate [51]; additionally, several genes that contribute to the regeneration of amputated limbs in axolotls have been already identified, providing information about how regeneration could be elicited in species with limited tissue repair [13,52]. Therefore, the identification of genes and molecular programs that are specifically regulated in the injured spinal cord of the axolotl (and that are also conserved in mammals) could provide information for the study of potential regenerative therapies in humans affected by this condition. 

The results obtained in our analysis show consistency with what was previously reported since the datasets used in this study were derived from the injured core tissue obtained from typical models of SCI induced in animals [9]; however, it is important to indicate that the nature of the sample obtained for analysis may influence the results. For example, it has been reported in rodents that the histopathology and gene expression profile in the injured spinal cord are not similar in the central zone than in the adjacent zone of injury [53,54]. Then, it would be interesting to study the changes in gene expression in the tissue adjacent to the lesion in the spinal cord of the axolotl and compare it with rodent models. On the other hand, in this study, the datasets used were obtained from adult animals, both neotenic axolotls and rodents, but some reports indicate that the regeneration capacity of axolotls decreases as age progresses [55]; thus, it would be important to analyze whether there are differences in the gene expression of the injured spinal cord between young and adult individuals. Lastly, another important consideration is that the expression patterns at the protein level may be different from what is observed at the transcript level, so it is important to study and compare the proteomics of SCI between axolotls and rodents to obtain a broader view of the regenerative mechanisms.

## 4. Conclusions

Through RNA-seq data analysis, we identified the molecular similarities and differences between rodents and axolotls after SCI. Immune-related pathways are upregulated in all studied species; however, a stronger immune response is noticeable at the level of gene expression in rodents in both acute and sub-acute periods of SCI. Differences were observed in genes related to self-renewal of stem cells (e.g., *Sox2* and *Tert*), which were upregulated during the first days of SCI in axolotls, with the same scenario for genes related to neurodevelopment, including *Foxa2*, *Vtn*, *Atf3*, *Atf6*, and *Cntf*. In axolotls, at 1 dpi, the genes classified as “response external stimulus” and “RNA processing” were linked through *Il6* and *Ddx1*. Although genes and molecular functions associated with neuron communication are suppressed in axolotl during SCI, it is only in rodents that we noticed the downregulation of processes related to sensory and motor functions. Thus, it is important to study in mammals the role of genes that are regulated in axolotls with SCI, to provide clues for the development of regenerative therapies.

## Figures and Tables

**Table 1:** Specifications of the RNA-seq datasets.

Species	Sequencing Platform	Type of Induced Injury	Samples	Reference
Axolotl	Illumina HiSeq 2500 paired end sequencing	The spinal cord was crushed at multiple levels, including the thoracic portion	Intact spinal cord, 1 dpi and 6 dpi	[15]
Rat	Illumina NovaSeq 6000 paired end sequencing	Lateral compression at the thoracic vertebrae T10 level	Intact spinal cord, 1 dpi and 7 dpi	[16]
Mouse	Illumina HiSeq 2000 paired end sequencing	Contusive injury at the thoracic vertebrae T9 level	Intact spinal cord, 2 dpi and 7 dpi	[17]

## Data Availability

All data used in this study are publicly available at https://www.ncbi.nlm.nih.gov/bioproject/ (accessed on 3 March 2023) under the following accession numbers: PRJNA378982, PRJNA760277 and PRJNA193596.

## Supplementary Materials

The following supporting information can be downloaded at: https://www.mdpi.com/article/10.3390/genes14122189/s1.
