# Supplementary material for: Transcriptome Profiling after Early Spinal Cord Injury in the Axolotl and Its Comparison with Rodent Animal Models through RNA-Seq Data Analysis
Source: Genes (Basel). 2023 Dec 8;14(12):2189. doi: 10.3390/genes14122189 (PMC10742908; doi:10.3390/genes14122189)
Supplement: Supplementary file 1 [file genes-14-02189-s001.zip › Supplementary figures.pdf]

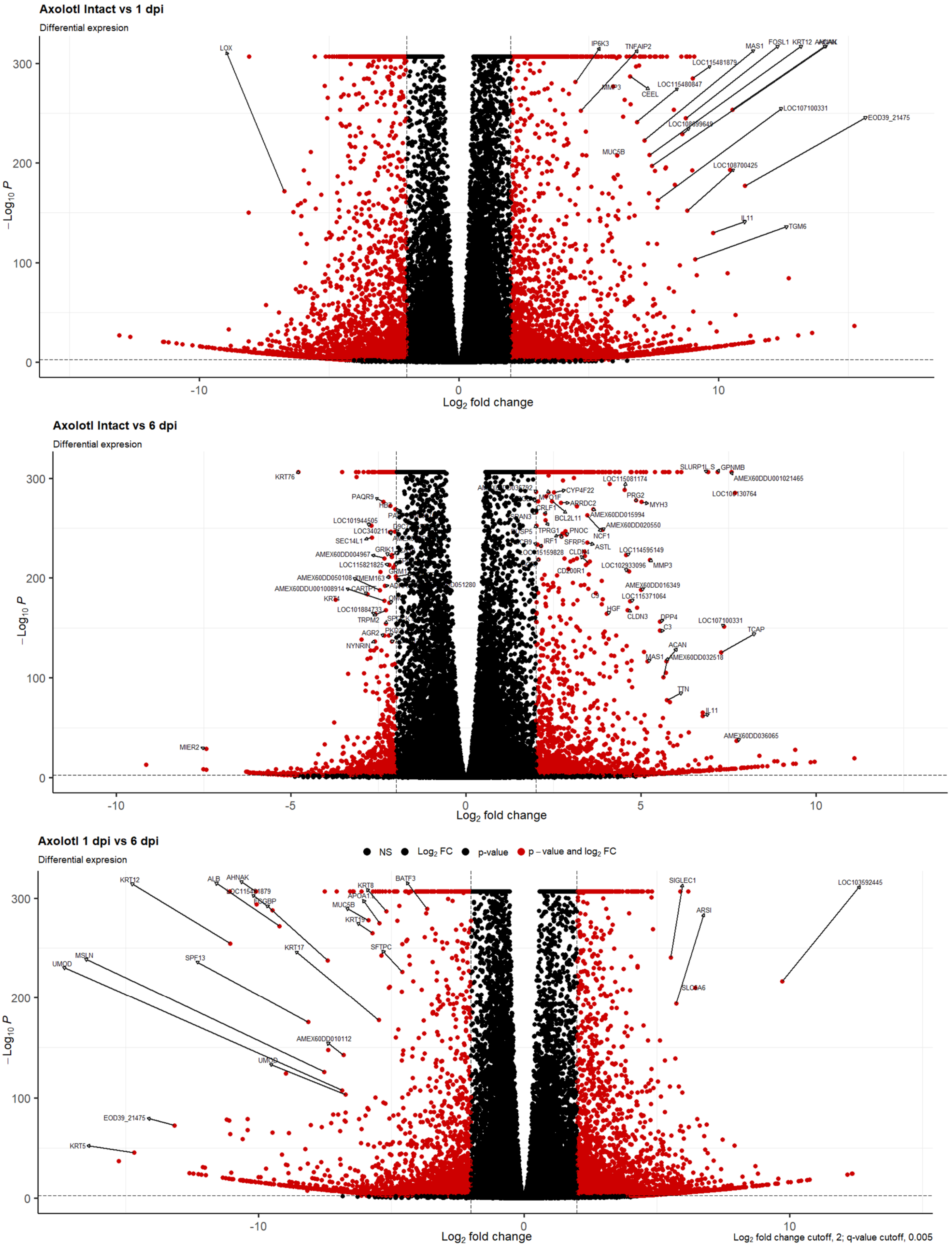

Supplementary Figure S1. Volcano plots showing significantly downregulated and upregulated genes in red dots (q-value <0.005) in axolotl: intact vs 1 dpi, intact vs 6 dpi, and 1 dpi vs 6 dpi. The axolotl present changes in larger number of genes after traumatic SCI, compared to the rat and mouse.

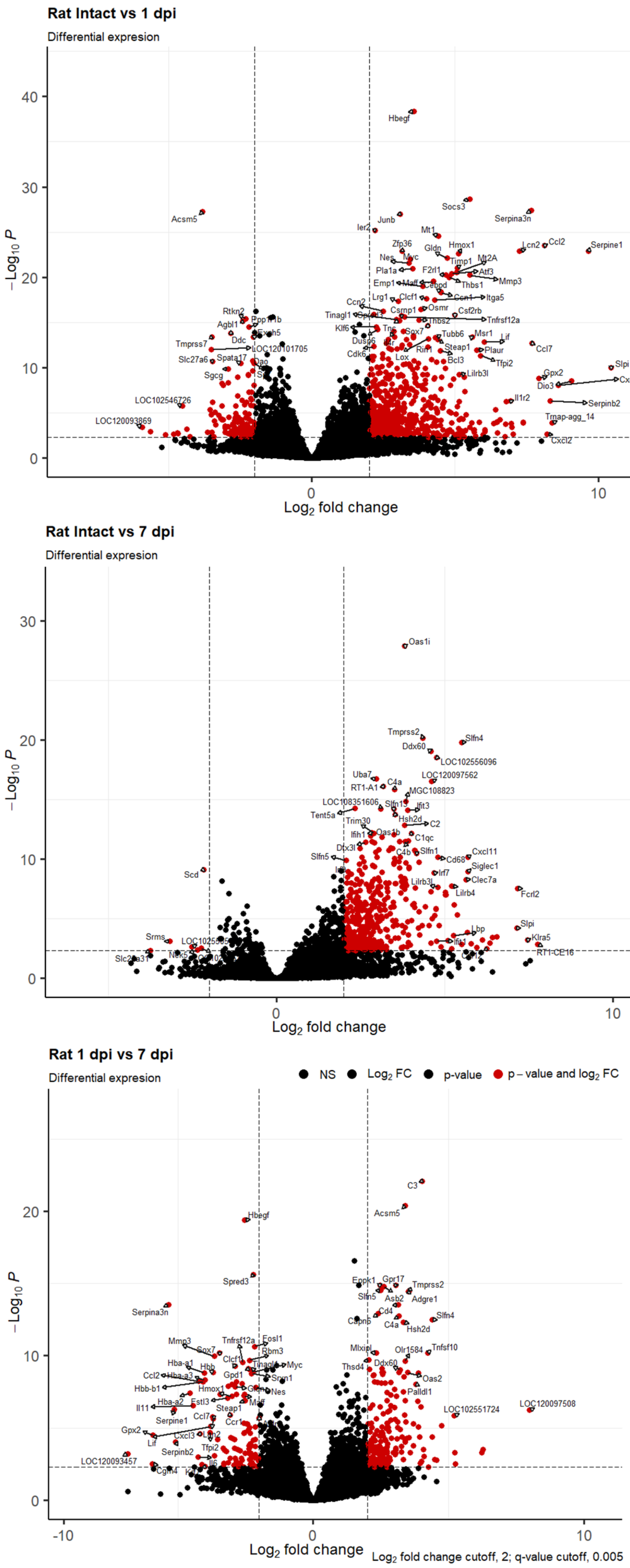

Supplementary Figure S2. Volcano plots showing significantly downregulated and upregulated genes in red dots (q-value <0.005) in rat: intact vs 1 dpi, intact vs 7 dpi, and 1 dpi vs 7 dpi.

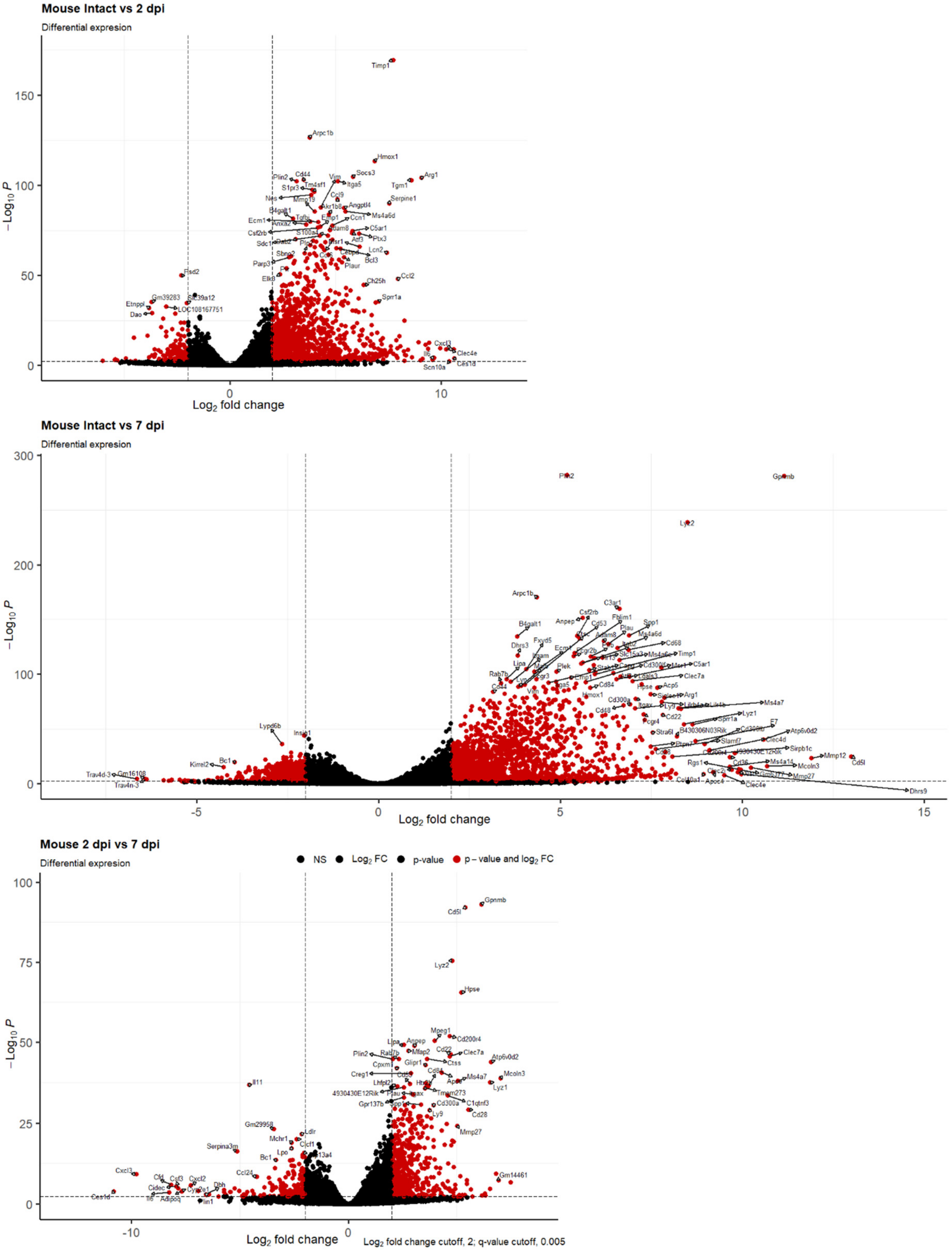

Supplementary Figure S3. Volcano plots showing significantly downregulated and upregulated genes in red dots (q-value <0.005) in mouse: intact vs 2 dpi, intact vs 7 dpi, and 2 dpi vs 7 dpi.

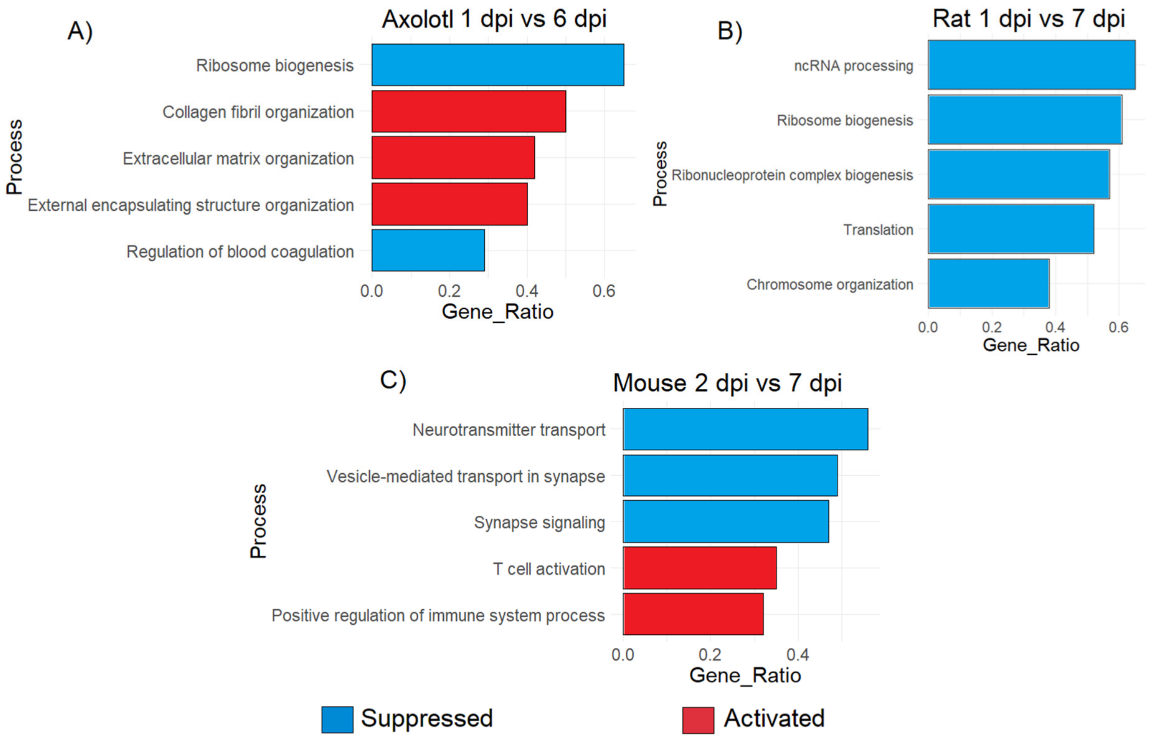

Supplementary Figure S4. GO enrichment analysis of biological processes in axolotl, rat, and mouse between acute and sub-acute SCI. Enriched biological processes for axolotl 1 dpi vs 6 dpi (A); rat 1 dpi vs 7 dpi (B); mouse 2 dpi vs 7 dpi (C). False discovery rate was estimated by BH correction. Cutoff for q-value was 0.005.

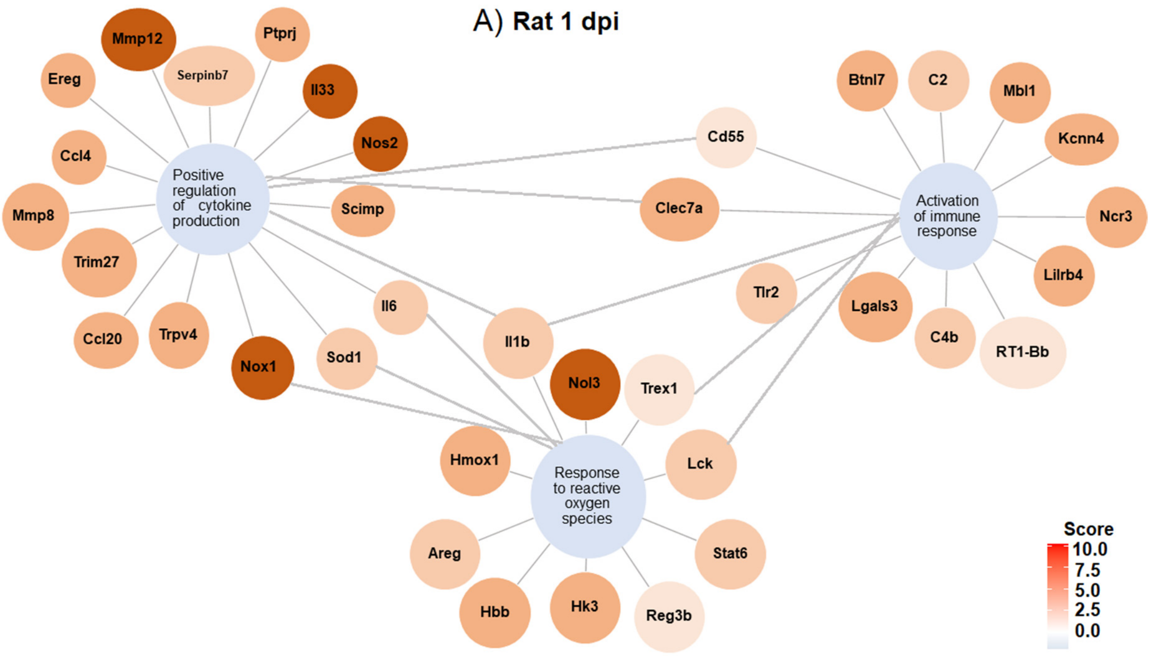

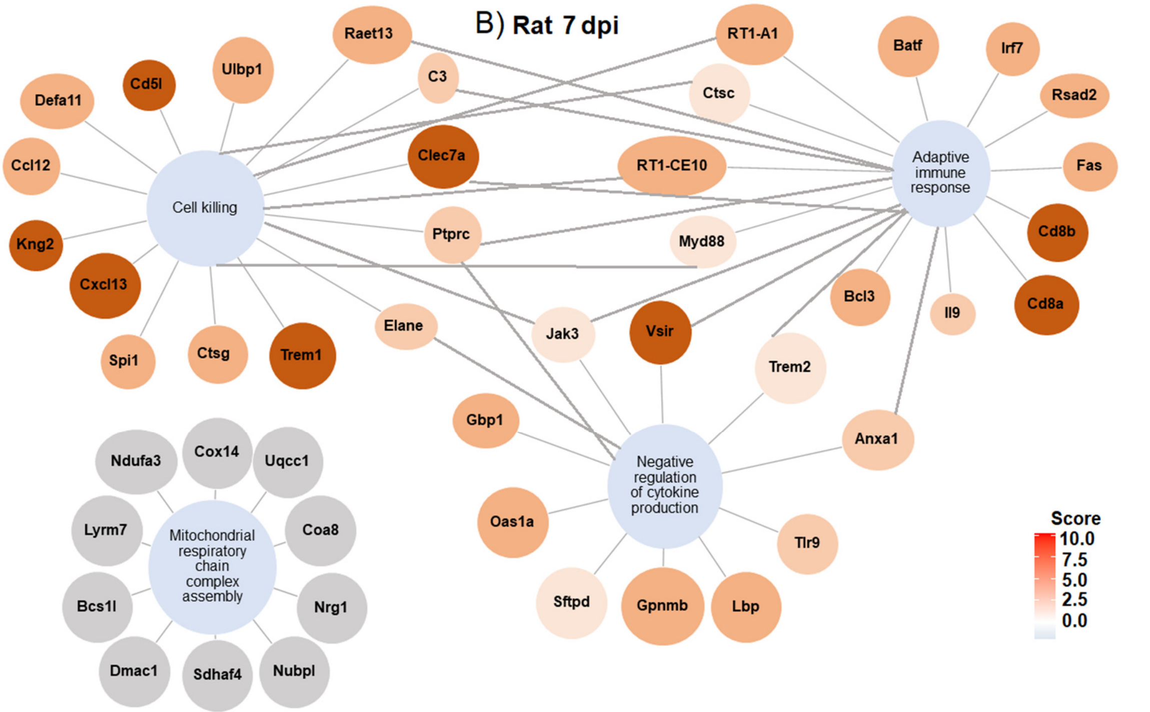

Supplementary Figure S5. Gene regulatory networks for GO terms in rat with SCI at 1 and 7 dpi. A) Gene networks for GO enriched terms “Positive regulation of cytokine production”, “Response to reactive oxygen species” and “Activation of immune response” at 1 dpi. B) Gene network for GO enriched terms “Cell killing”, “Adaptive immune response”, “Negative regulation of cytokine production” and “Mitochondrial respiratory chain complex assembly” at 7 dpi.

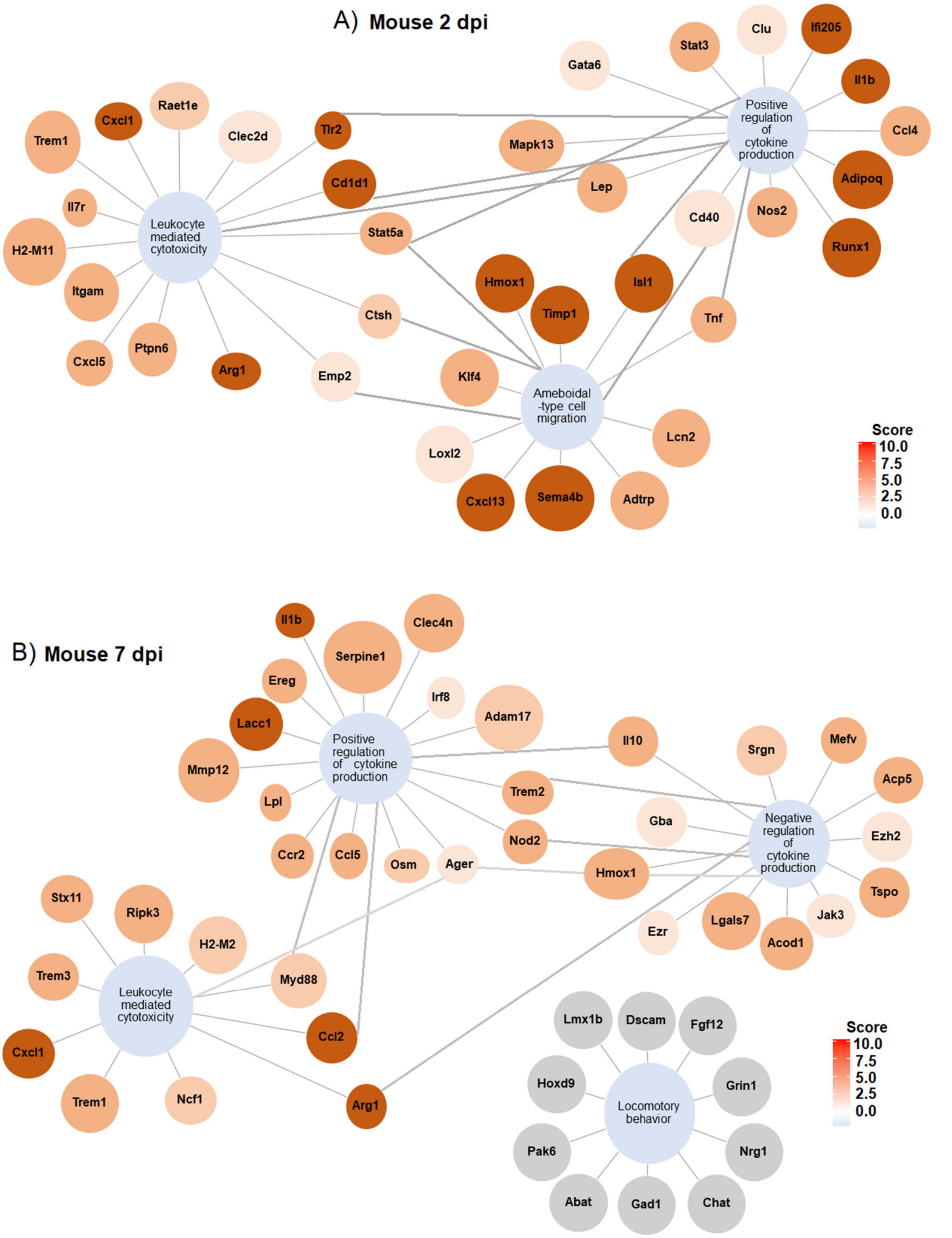

Supplementary Figure S6. Gene regulatory networks for GO terms in mouse with SCI at 2 and 7 dpi. A) Gene networks for GO enriched terms "Leukocyte mediated cytotoxicity", "Positive regulation of cytokine production" and "Ameboidal-type cell migration" at 2 dpi. B) Gene network for GO enriched terms "Leukocyte mediated cytotoxicity", "Positive regulation of cytokine production", "Negative regulation of cytokine production" and "Locomotory behavior" at 7 dpi.
